# α-Linolenic Acid and Risk of Heart Failure: A Meta-Analysis

**DOI:** 10.3389/fcvm.2021.788452

**Published:** 2022-01-04

**Authors:** Jiandi Wu, Min Qiu, Lichang Sun, Jiangxiong Wen, Dong-liang Liang, Sulin Zheng, Yuli Huang

**Affiliations:** ^1^Department of Cardiology, Affiliated Foshan Hospital, Southern Medical University, Foshan, China; ^2^Department of Cardiology, Shunde Hospital, Southern Medical University, Foshan, China

**Keywords:** α-linolenic acid, fatty acid, heart failure, risk, meta-analysis

## Abstract

**Background:** The α-linolenic acid is a plant origin n-3 fatty acid that may reduce the risk of cardiovascular disease. However, the effect of α-linolenic acid (ALA) on the risk of heart failure (HF) remains unclear. In this meta-analysis, we aimed to determine the role of ALA in the risk of incident HF.

**Methods:** Electronic databases were searched for studies up to August 10, 2021. Studies were included for meta-analysis if the adjusted risk of HF in different dietary intake or circulating levels of ALA was reported. We used the random-effects model to calculate the estimated hazard ratios (HRs) and 95% CI for higher ALA.

**Results:** A total of 6 studies (7 cohorts) comprising 135,270 participants were included for meta-analysis. After a median follow-up duration of 10 years, 5,905 cases of HF were recorded. No significant heterogeneity was observed among all the included studies. Random-effects model analyses showed that there was no significant association between ALA and the risk of incident HF, either assessed as quintiles (highest quintile vs. lowest quintile: HR = 0.95, 95% CI = 0.86–1.06) or per 1 *SD* increment (HR = 0.99, 95% CI = 0.95–1.01). Furthermore, we did not observe any association between ALA and the risk of HF in subgroup analyses performed according to age, sex, follow-up duration, and measuring method of ALA.

**Conclusions:** We found no association between ALA and the risk of incident HF, suggesting that ALA might not be effective in the prevention of HF.

## Introduction

Heart failure has become a growing global public health burden, with high rates of re-hospitalisation and mortality. Heart failure (HF) incidence increases dramatically with a growing ageing population, it was estimated that nowadays, there were more than 37.7 million HF patients worldwide (1). Although there have been significant improvements in the management of HF, the morbidity and mortality in HF patients were still high, which underscores the importance of the identification of novel risk factors and treatment modalities of HF (2–4).

Long-chain (LC) omega-3 polyunsaturated fatty acids (n-3 PUFA), including eicosapentaenoic acid (EPA) and docosahexaenoic acid (DHA), may play a protective role in the incidence and progression of HF, (5–7) through multiple mechanisms including anti-inflammation effects, improving cardiomyocytes' energy metabolism endothelial function (8). However, considering the sustainable supply and safety of the marine sources, plant-based n-3 PUFA is gaining much attention recently and is considered as an important alternative to n-3 PUFA sources (9). α-linolenic acid (ALA), a plant origin essential n-3 fatty acid, can be converted to EPA and DHA. Therefore, it is important to explore whether ALA has similar effects on the risk of HF. However, although previous studies showed that dietary patterns that included ALA-rich foods may reduce the risk of cardiovascular disease (10, 11), the association between ALA and HF was unclear. The Physicians' Health Study has found that dietary ALA intake level showed an inverse trend towards an association with HF, (12) while other studies did not find a similar association (13–15). To address the knowledge gap, we performed a pooled analysis of observational studies to generate data on the associations between ALA and the risk of incident HF.

## Methods

### Databases Search Strategy and Inclusion Criteria of Studies

We performed the meta-analysis following the recommendations of the Meta-analysis of Observational Studies in Epidemiology (MOOSE) group (16). Electronic databases, including PubMed, Google scholar, Cochran Library, and EMBASE, were searched for studies from inception until 31 July 2021, using a combined text and medical subheadings (MeSH) search strategies, with multiple terms related to “Omega-3 PUFA” or “α-linolenic acid” and “heart failure”. We were restricted to human studies. Reference lists of the related articles and included studies were manually reviewed to identify potentially missed pieces of research. The detailed strategy used for searching PubMed is presented in Supplementary File 1. When searching other databases, the strategies were similar.

We included observational studies for meta-analysis if they met the following criteria: (1) observational studies (including cohort studies, nest case-control studies, and *post hoc* analyses of randomised controlled trials) with adult participants (aged ≥18 years); (2) dietary intakes or circulating levels of ALA were detected; (3) the risk of HF associated with different levels of ALA were reported.

We excluded studies if: (1) they were cross-sectional studies; (2) the risk of HF was unadjusted for confounders; (3) the follow-up duration was <1 year; (4) duplicated articles were reported from the same cohort dataset. If more than one report used data from the same participants' cohort, only the latest published article was included.

### Data Extraction and Quality Assessment of the Included Studies

Two investigators (J.W. and M.Q.) independently conducted article searches and screened the retrieved articles. Full copies of potentially suitable studies were reviewed. Key information of the included studies, such as country, cohort characteristics, methods for measurement of ALA, study sample, sex proportion, mean/median age, HF cases, and follow-up duration was recorded. If the essential message was not reported, we contacted the principal authors for clarification.

The Newcastle–Ottawa Quality Assessment Scale for observational cohort studies was used to determine the study quality (17), which is based on: the selection (3 items with 1 point each), comparability (1 item with up to 2 points), and exposure/outcome (3 items with 1 point each), with the total score from 0 to 9. Included studies were classified as good quality (≥7 points), fair quality (4–6 points), or poor (<4 points), respectively (18, 19).

### Data Synthesis

The outcome evaluated in this analysis was the risk of incident HF associated with different levels of dietary intake or circulating ALA. We extracted outcome data which adjusted for the maximal number of potential confounders for analysis. In the included studies, the associations of the HF risk and ALA levels were reported in different ways, such as per quartiles or quintiles of ALA levels, or per 1 *SD* increment in the continuous trait. Therefore, we used two methods to transfer the data into consistent comparisons. We compared the hazard ratio (HR) of HF risk in participants with the highest quintile of ALA with those with the lowest quintile and calculated the HR as per 1 *SD* increment in ALA. If effect measures were not reported as per quintile or per 1 *SD* change in the included studies, we converted the data following the previously published methods (20). Briefly, we assumed that the exposure variable (ALA) was with normal distribution and its association with the interesting outcome (risk of HF) was log-linear. The expected difference in means of the highest vs. lowest tertile is 2.18 *SD*s, and 2.54 *SD*s for the highest vs. lowest quartile, 2.8 *SD*s for the highest vs. lowest quintile, respectively (20). Therefore, HR reported as top vs. bottom quintile comparison can be converted to per 1 *SD* change by using a division conversion factor of 2.8 to the log HR. Accordingly, HRs for comparisons of extreme tertiles and quartiles are divided by 2.18 and 2.54, respectively. Conversely, HRs and 95% CIs reported as comparisons of quartiles or tertiles can be converted to comparisons of quintiles, by using a multiplication conversion factor of 2.8/2.54 or 2.8/2.18 to the log HRs.

The inverse variance approach was used to calculate the log HRs and corresponding SEs with the random-effects models (21). The I^2^ statistics were used to test heterogeneity. An I^2^ value > 50% was regarded as with significant heterogeneity among the included studies (2). Subgroup analyses of the primary outcome were conducted according to sex, age, follow-up period, and measure of ALA. We evaluated the publication bias by inspecting the funnel plots, as well as by using Egger's test and Begg's test. Finally, we performed the sensitivity analyses by changing the random-effects model into the fixed-effects model in the pooled analysis. We also recalculated the HRs by excluding one study at a time to determine the effect of every study on the pooled risk.

All analyses were performed using Stata 15.0 (StataCorp LP, College Station, TX, USA) and RevMan 5.3 (The Cochrane Collaboration, Copenhagen, Denmark). All *P*-values are two-tailed, and a *P* < 0.05 is considered as with statistical significance.

## Results

### Studies Characteristics and Quality

**Figure 1:**
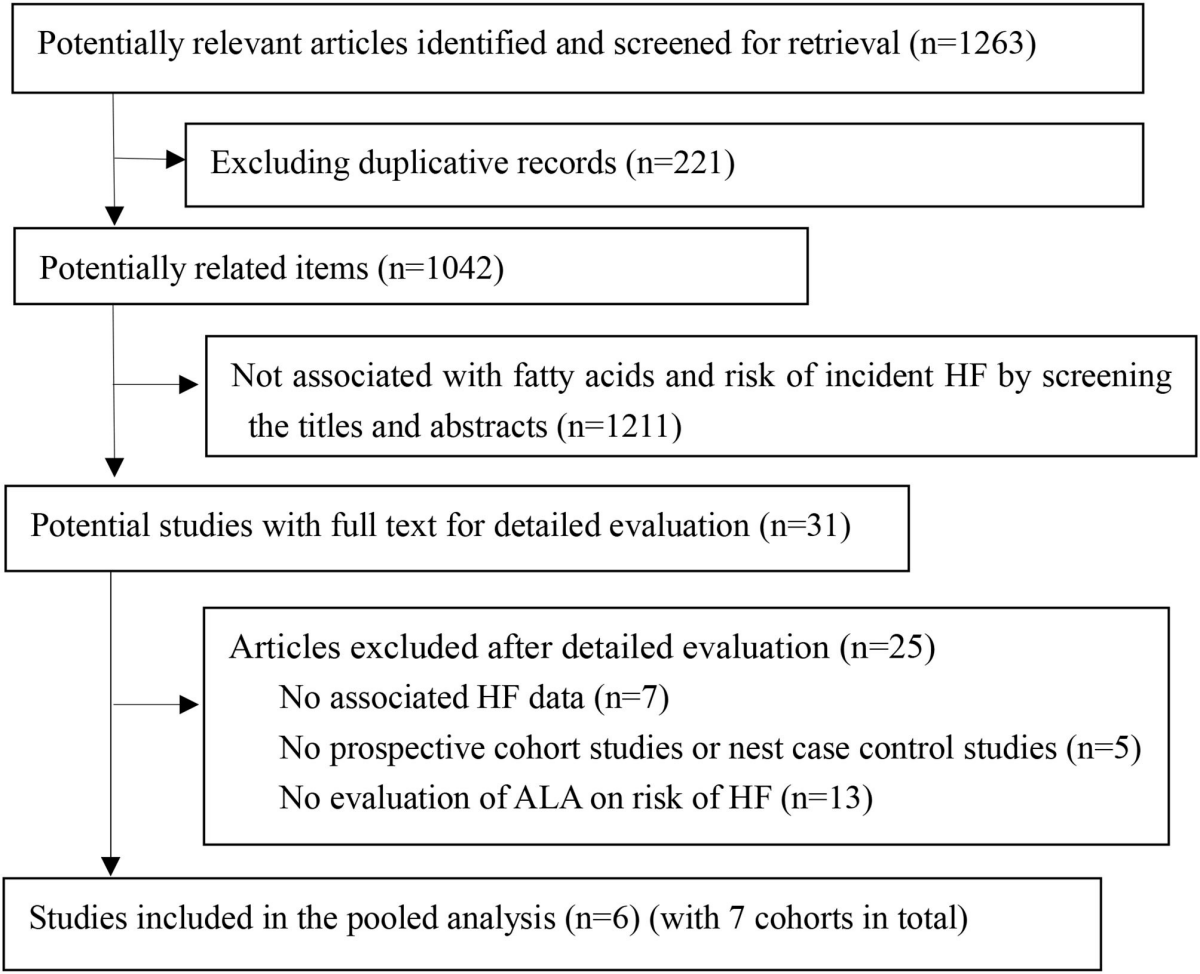
The flow of papers through review. ALA, α-Linolenic acid; HF, heart failure.

We retrieved 1,263 article items from the searched electronic databases. After removing duplication records, two authors independently screened the titles and abstracts. Thirty-one articles were fully evaluated, and 6 studies were included in the pooled analysis finally (Figure 1). In the included studies, two of them reported dietary intake of ALA (14, 22), 3 reported circulating ALA level (12, 15, 23), and only one study reported both dietary and circulating ALA level (13). The 6 studies (7 cohorts) included 135,270 participants for analysis, with their main characteristics resented in Table 1. One study only enrolled men, two studies only enrolled women, while all other studies enrolled both genders. After the median follow-up duration of 10 years (ranged from 8.4 to 14.3 years), 5,905 cases of HF were recorded. The adjusted confounders for HF risk in the pooled studies are presented in Supplementary File 5. Only one study was graded as fair, and all other 5 studies were considered as of good quality according to our predefined quality assessment criteria (Supplementary File 3).

**Table 1:** Characteristics of studies on ALA and risk of HF.

**Study**	**Country/Region**	**Cohort characteristics**	**Measure of ALA (method)**	**Sample size (%women)**	**HF case (n)**	**Age (y), average (range or SD)**	**Follow-up (y)**
Djousse et al. (23)	United States	Community residents	Serum (GC)	2003 (61.2%)	655	77.7 (4.4)	9.7
Wilk et al. (12)	United States	Healthy male physicians	Plasma Phospholipid (GC)	1576 (0%)	786	58.7 (NA)	8.4
Lemaitre et al. (13)	United States	Community residents	Dietary (FFQ)	4432 (NA)	1072	≥65 y (NA)	11.9
Lemaitre et al. (13)	United States	Community residents	Plasma Phospholipid (GC)	2957 (63.2%)	686	72.1 (5.2)	10.4
Belin et al. (14)	United States	Postmenopausal women	Dietary (FFQ)	84493 (100%)	1858	63.3 (50-79)	10.0
Levitan et al. (22)	Sweden	Swedish women	Dietary (FFQ)	36234 (100%)	651	61.6 (48-83)	9.0
Yamagishi et al. (15)	United States	Community residents	Plasma Phospholipid (GLC)	3575 (53.4%)	197	53.7 (5.6)	14.3

*ALA, α-linolenic acid; ARIC, Atherosclerosis Risk in Communities; FFQ, food frequency questionnaire; GC, gas chromatography; GLC, gas-liquid chromatogram; HF, heart failure; NA, not available*.

### ALA and Risk of Incident HF

**Figure 2:**
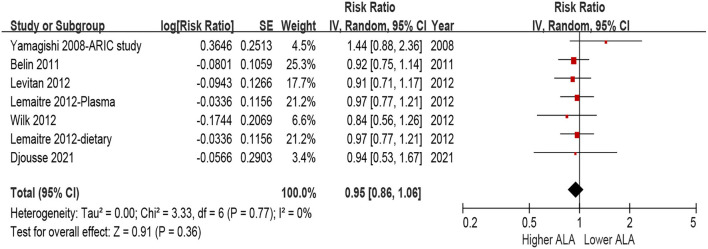
Forest plot for risk of HF associated with ALA level by quintiles. ALA, α-Linolenic acid; CI, confidence interval; HF, heart failure.

**Figure 3:**
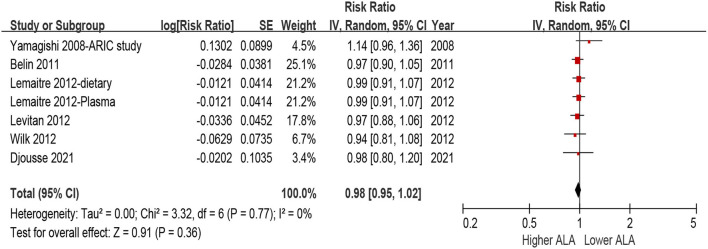
Forest plot for risk of HF associated with ALA level by per 1 SD increment. ALA, α-Linolenic acid; CI, confidence interval; HF, heart failure.

No statistical heterogeneity observed among all the included studies for the association between ALA and incident HF (I^2^ = 0%, *P* = 0.77). Random-effects model analyses showed that there was no significant association between ALA and risk of incident HF, either assessed as quintiles (Figure 2, highest quintile vs. lowest quintile: HR = 0.95, 95% CI = 0.86–1.06, *P* = 0.36) or per 1 *SD* increment (Figure 3, HR = 0.99, 95% CI = 0.95–1.01, *P* = 0.36). We did not find any evidence of publication bias by the funnel plot (Supplementary Files 4, 5), Egger's or Begg's tests (both *P* > 0.1).

### Sensitivity Analyses and Subgroup Analyses

We conducted several sensitivity analyses to confirm the HF outcome was consistent by using the fixed-effects models instead of random-effects models for meta-analysis or recalculating the estimated HRs and 95% CI by excluding one study at each time. Furthermore, in subgroups performed according to age, sex, follow-up duration, and measuring method of ALA, we did not observe any significant association between ALA levels and the risk of incident HF. Furthermore, no significant heterogeneity was observed among all subgroup comparisons (all I^2^ = 0%, *P* ≥ 0.48, Table 2).

**Table 2:** Subgroup analyses of the association between ALA and risk of HF.

	**Studies (N)**	**Quintile 5 vs. Quintile 1**		**Per 1 SD increment**	
		**HR (95%CI)**	*P* value^*^/I^**2**^	**HR (95%CI)**	*P* value^*^/I^**2**^
**Average age (years)**					
<65	4	0.94 [0.81, 1.10]	0.85/0%	0.99 [0.93, 1.04]	0.85/0%
≥65	3	0.97 [0.83, 1.13]		0.98 [0.93, 1.03]	
**Sex**					
Female	2	0.92 [0.78, 1.08]	0.69/0%	0.97 [0.92, 1.03]	0.68/0%
Male	1	0.84 [0.56, 1.26]		0.94 [0.81, 1.08]	
**Follow-up duration (years)**					
<10 years	3	0.90 [0.74, 1.09]	0.48/0%	0.96 [0.90, 1.03]	0.48/0%
≥10 years	4	0.98 [0.86, 1.10]		0.99 [0.95, 1.04]	
**Measure of exposure**					
Dietary	3	0.92 [0.81, 1.04]	0.51/0%	0.98 [0.94, 1.02]	0.68/0%
Circulating	4	0.99 [0.83, 1.18]		1.00 [0.94, 1.06]	

* *For heterogeneity among subgroups. ALA, α-linolenic acid; CI, confidence intervals; HF, Heart failure; HR, hazard ratios; SD, standard deviation*.

## Discussion

To the best of our knowledge, this is the first pooled analysis evaluating the association between ALA and the risk of incident HF. We did not find any association between ALA (neither detected by dietary intake nor circulating level) and the risk of HF. The null result was further supported by no heterogeneity observed among multiple subgroup comparisons.

Basic research on ALA on cardiac function had yielded inconsistent results. In a mice/rat model with doxorubicin-mediated cardiotoxicity, the prophylactic administration of the ALA was cardioprotective by decreasing inflammation, apoptosis, and mitochondrial dysfunction (24, 25). Enrichment of ALA in rodent diet reduced oxidative stress and inflammation, resulted in less left ventricle dilation, and decreased myocardial fibrosis during myocardial infarction (26, 27). In diabetic or obese mice, dietary ALA counters cardioprotective dysfunction mainly through anti-inflammatory effects (28, 29). ALA may also confer beneficial anti-platelet and anti-inflammatory effects, and interaction with gut microbiota, which is significantly associated with the risk HF (20, 30). However, these effects were not observed in a rat model under pressure overload conditions (31).

In humans, evidence suggests that increasing ALA is associated with favourable cardiometabolic status and lower inflammation status (32, 33), which may play a protective role in cardiac function. Data from NHANES found that ALA and DHA can enhance the negative association between maximal oxygen uptake and C-reactive protein, suggesting that the anti-inflammatory response to maximal oxygen uptake capacity is related to levels of ALA and (34). However, in a randomised clinical trial, it was also found that increasing plasmatic ALA had no impact on plasma inflammatory biomarkers (35). Taking these studies with our results together indicates that humans may not benefit from ALA intake in the view of prevention of HF.

In contrast, marine n-3 PUFA had been shown to be protective for HF (12, 13, 36). In humans, ALA can be bioconverted into EPA and DHA, however, the rate is not sufficiently. The rate of bioconversion from ALA to DHA is about 0.05–0.5%, and to EPA is 0.2–10% (9). The limited bioconversion rate may limit the potential cardioprotective effects. Furthermore, this conversion rate from ALA to EPA/DHA was influenced by sex and genetic factors (especially for variants of desaturase and elongase enzymes), as well as dietary intake level of EPA/DHA. In a Danish cohort with 53,909 participants followed for a median of 13.4 years, an inverse association between dietary ALA intake and the risk of CVD was observed in individuals with a low intake of marine n-3 PUFA, while not in those with a higher intake of marine n-3 PUFA. These findings suggested that ALA may be associated with a lower risk of CVD only in those individuals with not adequate intake of marine n-3 PUFA (37). However, whether the relationship between ALA and the risk of incident HF was similar remains unexplored.

Our study has several major strengths. First, most of the included studies were adjusted for multiple confounders, including traditional cardiovascular risk factors and energy intake. This may mitigate the effect of potential confounders influencing the results. Second, the associations were consistently determined by category (highest quintile *vs*. lowest quintile) or continuous (per 1-*SD*) increment of ALA. Furthermore, no heterogeneity was observed among the included studies (I^2^ = 0%) and pooled results were also consistent among comprehensive subgroup analyses. However, some limitations of the current study should be discussed. First, most of the included studies were from the United States, and only one study was from Sweden. The association between ALA and incident HF was still unclear in other populations, especially those with different dietary patterns. Second, we performed this meta-analysis based on the study level. No individual participant data were available, so residual bias cannot be totally avoided. Third, the cardio-protective effects of ALA may be modified by different intakes of LC n-3 PUFAs (37). However, only two studies adjusted the level of DHA and EPA in the analysis. Finally, only one measurement of ALA at baseline was detected in most of the included studies, and the change of ALA overtime was not accounted for. However, the Physicians' Health Study showed that the use of single baseline level ALA, or mean level of between baseline and long-term follow-up measures (up to 15 years), yielded similar associations (both null) on their relationship with HF (38).

## Conclusions

We did not find any association between ALA (measured either from dietary questionnaires or with a circulating biomarker) and the risk of incident HF. These results suggest that plant origin ALA cannot be regarded as a substitute for LC n-3 fatty acids in the viewpoint for the prevention of HF. Further studies with other populations (e.g., Asians, Africans) are needed to determine whether a high intake of ALA can prevent HF.

## Data Availability Statement

The original contributions presented in the study are included in the article/Supplementary Material, further inquiries can be directed to the corresponding author/s.

## Author Contributions

JWu, MQ, LS, D-lL, JWe, and YH: research idea and study design. JWu, MQ, and LS: data acquisition. JWu and MQ: data analysis and interpretation. JWu and LS: statistical analysis. JWu and YH: supervision and mentorship. All authors contributed important intellectual content during manuscript drafting or revision and accept accountability for the overall work by ensuring that questions pertaining to the accuracy or integrity of any portion of the work are appropriately investigated and resolved.

## Funding

This project was supported by the Guangdong Basic and Applied Basic Research Fund (Key Project of Guangdong-Foshan Joint Fund) (2019B1515120044), Science and Technology Innovation Project from Foshan, Guangdong (FS0AA-KJ218-1301-0006), and the Clinical Research Startup Program of Shunde Hospital, Southern Medical University (CRSP2019001). The funders had no role in study design, data collection, data analysis, data interpretation, or writing of the report.

## Conflict of Interest

The authors declare that the research was conducted in the absence of any commercial or financial relationships that could be construed as a potential conflict of interest.

## Publisher's Note

All claims expressed in this article are solely those of the authors and do not necessarily represent those of their affiliated organizations, or those of the publisher, the editors and the reviewers. Any product that may be evaluated in this article, or claim that may be made by its manufacturer, is not guaranteed or endorsed by the publisher.
