# Supplementary material for: α-Linolenic Acid and Risk of Heart Failure: A Meta-Analysis
Source: Front Cardiovasc Med. 2022 Jan 4;8:788452. doi: 10.3389/fcvm.2021.788452 (PMC8764440; doi:10.3389/fcvm.2021.788452)
Supplement: Supplementary file 1 [file Data_Sheet_1.doc]

**Supplementary file 1. Literature search strategy for Pubmed**

| **#1** | ("Fatty Acids, Omega-3"[Mesh]) OR "α-linolenic acid"[Mesh] |
| --- | --- |
| **#2** | ((((((((("n-3 fatty acids"[Text Word]) OR "omega-3 fatty acids"[Text Word]) OR ω-3 fatty acids [Text Word]) OR "n-3 fatty acid"[Text Word]) OR "omega-3 fatty acid"[Text Word]) OR "ω-3 fatty acid"[Text Word]) OR "α-linolenic acid"[Text Word]) OR ALA [Text Word]) OR "polyunsaturated fatty acid" [Text Word]) OR "polyunsaturated fatty acids" [Text Word] OR "PUFA" [Text Word] |
| **#3** | #1 OR #2 |
| **#4** | Heart failure [Mesh] |
| **#5** | (((((("Heart failure"[Text Word]) OR " Cardiac Failure "[Text Word]) OR " Myocardial Failure "[Text Word]) OR " Cardiac dysfunction"[Text Word] OR " Heart dysfunction "[Text Word]) OR " Myocardial dysfunction "[Text Word]) |
| **#6** | #4 OR #5 |
| **#7** | #3 AND #6 |
| **#8** | animals[MeSH Terms] |
| **#9** | humans[MeSH Terms] |
| **#10** | #8 NOT #9 |
| **#11** | #7 NOT #10 |

**Supplementary file 2**. **Confounders adjusted in the included studies**

| **Study** | **Confounder adjusted** |
| --- | --- |
| Djousse 2021 | Age, sex, race, field center, education, serum albumin, BMI, waist circumference, eGFR, physical activity, alcohol, smoking, hormone replacement therapy, unintentional weight loss, and other nonesterified fatty acid |
| Wilk 2012 | Age, AF, hypertension, BMI, alcohol, smoking, exercise. |
| Lemaitre 2012 | Age, sex, enrollment site, race, education, smoking, BMI, waist circumference, alcohol consumption, and total energy intake |
| Belin 2011 | Age, ethnicity, education, physical activity, smoking, alcohol, DM, hypertension, AF, MI/CABG/PTCA, BMI, time-dependent MI, fiber, fruit/vegetable servings, saturated fat intake, DHA+EPA |
| Levitan 2010 | Age, BMI, physical activity, energy intake, alcohol consumption, fibre consumption, Na consumption, education, family history of MI, cigarette smoking, living alone, postmenopausal hormone use, self-reported history of hypertension, and self-reported history of high cholesterol. |
| Yamagishi 2008-ARIC study | Age, sex, BMI, SBP, antyhypertensive medication use, total and HDL-C, DM, smoking status, cigarette-years, ethanol and energy intake, education level and sports index. |

AF: atrial fibrillation; BMI: body mass index; CABG: coronary artery bypass grafting; DM: diabetes mellitus; eGFR: estimated glomerular filtration rate; HDL-C: high-density lipoprotein cholesterol; MI: myocardial infarction; PSBP: systolic blood pressure

**Supplementary file 3. Quality Assessment of the included studies**

| **Study** | **Selection (stars awarded)** | **Comparability (stars awarded)** | **Outcome (stars awarded)** | **Quality (total stars awarded)** |
| --- | --- | --- | --- | --- |
| Djousse 2021 | 3 | 2 | 3 | Good (8) |
| Wilk 2012 | 2 | 1 | 3 | Fair (6) |
| Lemaitre 2012 | 3 | 2 | 3 | Good (8) |
| Belin 2011 | 3 | 2 | 3 | Good (8) |
| Levitan 2010 | 3 | 2 | 3 | Good (8) |
| Yamagishi 2008-ARIC study | 3 | 2 | 3 | Good (8) |

ARIC: Atherosclerosis Risk in Communities


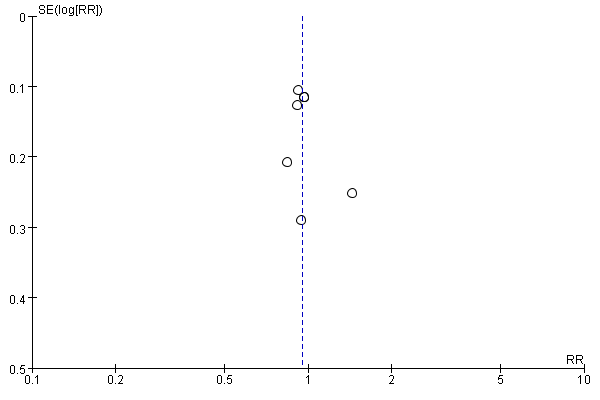


**Supplementary File 4. Funnel plot for evaluation of publication bias in studies evaluated the association between ALA (by Quintile) and risk of HF**

ALA: α-linolenic acid; HF: heart failure


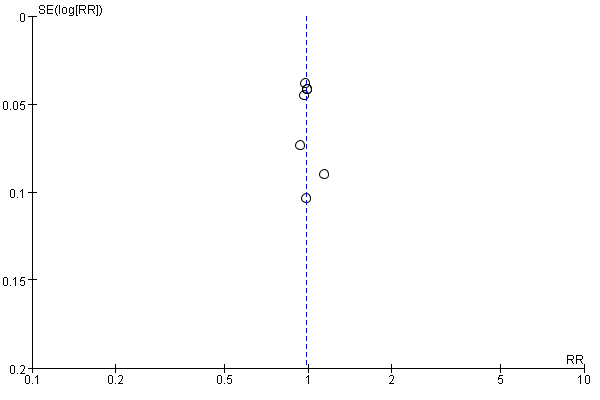


**Supplementary File 5. Funnel plot for evaluation of publication bias in studies evaluated the association between ALA (by per SD increment) and risk of HF**

ALA: α-linolenic acid; HF: heart failure
